# Sex-Specific Effect of Recalled Parenting on Affective and Cognitive Empathy in Adulthood

**DOI:** 10.1007/s12144-015-9405-z

**Published:** 2016-01-18

**Authors:** Minna T. Lyons, Gayle Brewer, Emily J. Bethell

**Affiliations:** 1 School of Psychology, University of Liverpool, Bedford Street South, Liverpool, L69 7ZA UK; 2 University of Central Lancashire, Preston, England; 3 Liverpool John Moores University, Liverpool, UK

**Keywords:** Affective and cognitive empathy, Parental care, Parental overprotection, Sex differences

## Abstract

Previous research has demonstrated the influence of parenting on the development of children’s empathy. However, few studies have considered the impact of parents on empathy in adulthood, specific components of empathy, or the importance of parent and child biological sex. In the present study, 226 participants (71 men) completed online versions of the Parental Bonding Instrument (Parker et al. *British Journal of Medical Psychology, 52,* 1–10 1979), Empathy Quotient (Baron-Cohen and Wheelwright *Journal of Autism and Developmental Disorders, 34,* 163–175 2004), and Interpersonal Reactivity Index (Davis *JSAS Catalog of Selected Documents in Psychology, 10,* 85 1980). Paternal care and overprotection influenced affective empathy in men, whilst maternal overprotection predicted affective empathy in women. Further, maternal care related to cognitive empathy in men, whilst none of the parental care variables related to cognitive empathy in women. Findings are discussed in relation to sex differences in childhood parenting experiences on adult cognitive and affective empathy.

## Introduction

Empathy is a key socio-emotional skill which allows us to infer what others may be thinking or feeling, allowing us to adapt our own behaviour accordingly (Baron-Cohen and Wheelwright 2004). It is a multi-dimensional construct, consisting of affective and cognitive components (Shamay-Tsoory et al. 2009). Affective empathy is characterised as an emotional response to the emotional states of others, whereas cognitive empathy is the ability to understand, and take a non-egocentric perspective to others’ emotions (Baron-Cohen and Wheelwright 2004). Emotional and cognitive empathy are associated but separate constructs (Reniers et al. 2011) which relate differently to behavioural outcomes (such as altruistic sharing; Edele et al. 2013) and have distinct neural correlates (Moore et al. 2015).

Attachment theorists emphasise the importance of sensitive parenting for children’s development of empathy and other socio-emotional skills (Sroufe 2005). By providing a secure supportive base and encouragement to explore the environment, the child has opportunity to divert attention away from the self, which allows other-focused abilities to develop. It is therefore likely that childhood experience with parents contributes to the individual differences seen in both affective and cognitive empathy in adulthood. According to Parker et al. (1979), parenting consists of two components: care (i.e., warmth and affection) and overprotection (i.e., intrusiveness and control). Although it is not clear how care and overprotection may function together in empathy development, a study by Gao et al. (2010) found that low maternal care and low paternal overprotection were important predictors of emotional detachment factor of psychopathy. Thus, it is possible that high care and high overprotection together form an important combination that influences empathy development. Despite research demonstrating the importance of parental care in empathy development in childhood (e.g., Richaud de Minzi 2013; Strayer and Roberts 2004) and adolescence (e.g., Batanova and Loukas 2012), there is relatively little research looking at parental influence later in adulthood.

In childhood, for example, affective empathy has been linked to parental warmth (Zahn-Waxler 1991), while in adolescence, both affective and cognitive empathy have been associated with maternal support (Soenens et al. 2007). Mothers and fathers have differential influence on the socio-emotional development in boys and girls. Batanova and Loukas (2012) found that in girls rather than in boys, parent-child conflict related to low cognitive empathy. Maternal care, on the other hand, may be more important for socio-emotional development and perspective taking in boys rather than girls (Etzion-Carasso and Oppenheim 2000; Laranjo et al. 2010). The effect of each parent for empathy development in men and women, separately, is therefore worth considering.

A small body of research suggests it is worth testing for a possible effect of parental care and overprotection on empathy development into adulthood. One longitudinal study found that empathic concern (a component of affective empathy) at the age of 31 was predicted by paternal involvement and maternal tolerance of dependency at the age of 5 (Koestner et al. 1990). In addition, a retrospective study reported that recalled parental care in childhood was associated with empathic concern, and overprotection was associated with perspective taking in adulthood (Britton and Fuendeling 2005). Previous research also indicates that poor quality parent-child relationship is related to a range of outcomes in adulthood, including the development of personality traits and behaviours characterised by low empathy (e.g., Gao et al. 2010; Jonason et al. 2014; Lyons et al. 2013). In addition, behaviours in adulthood that are linked to high empathy (e.g., cooperation; Artinger et al. 2014), in other studies have been found to be associated with high-quality parental care during childhood (Josefsson et al. 2013). However, studies have not yet addressed how parental care and overprotection affect both cognitive and affective empathy in adulthood, and what role the sex of the child and the parent may play in this.

The present study should be considered as exploratory, aiming to add to the existing literature by using an adult sample, and considering (i) the multi-dimensional construct of empathy, and (ii) the possibility for a domain-specific effect of each parent, depending on the sex of the offspring.

## Method and Materials

Men (*N* = 71) and women (*N* = 155) aged 18–62 years (*M* = 26.54, *SD* = 8.83) were recruited via online research forums, adverts at two Universities in the UK, and social networking sites for an online study investigating “childhood experiences and personality”. Criteria for taking part were that participants were at least 18 years old and had grown up in a two-parent household. After providing informed consent, participants completed a series of online measures assessing parental bonding and empathy, followed by an on-line debrief.

The Parental Bonding Instrument (PBI: Parker et al. 1979) is a 50 item retrospective measure of perceived parenting style experienced in childhood. Items relating to maternal (α = .93) and paternal (α = .94) care (e.g., “*was affectionate to me*”), and maternal (α = .89) and paternal (α = .88) overprotection (e.g., “*tried to make me feel dependent on her*/*him*”) were rated on a four point scale (0 = “*very unlike*”, to 3 = “*very like*”). The PBI demonstrates internal consistency (Wilhelm and Parker 1990), long-term test-retest reliability (Wilhelm et al. 2005) and has been validated for use in a range of populations and cultures (Narita et al. 2000).

The Empathy Quotient (EQ; Baron-Cohen and Wheelwright 2004) is a 40 item measure of affective and cognitive empathy answered on a four point scale (from “*strongly disagree*” to “*strongly agree*”). Non-empathic responses are scored as 0, and the empathic responses scored either 1 or 2, depending on the strength of the response. Consistent with Muncer and Ling (2006) affective empathy (α = .57, e.g. “*Seeing people cry does not really upset me*”) and cognitive empathy (α = .80, e.g. “*I can tell if someone is masking their true emotions*”) scales were employed. Previous research (e.g. Allison et al. 2011; Lawrence et al. 2004) has established the reliability and validity of the measure.

The Interpersonal Reactivity Index (IRI; Davis 1980) contains 28 items, answered on a five point scale (0 = “*does not describe me well*” to 4 = “*describes me very well*”)*.* In the present study, we include two of the four empathy subscales: empathic concern (α = .82) and perspective taking (α = .75) as measures for affective and cognitive empathy, respectively. Example items include “*I often have tender*, *concerned feelings for people less fortunate than me*” (empathic concern) and “*I sometimes try to understand my friends better by imagining how things look from their perspective*” (perspective taking). The measure has been validated for use in a range of cultures and contexts (De Corte et al. 2007; Ferndandez et al. 2011; Peloquin and Lafontaine 2010).

We used both the IRI and EQ scales in line with previous studies that used one or other, or both questionnaires (e.g. Maurage et al. 2011). In our study IRI scores were significantly correlated with the EQ cognitive (*r* = .40, *p* < .001) and affective (*r* = .67, *p* < .001) subscales, so to avoid issues of colinearity we summed and averaged z-scores to produce a single variable for each empathy component.

## Results

We report the descriptive statistics and cross-correlations in Table 1. Maternal care scale had a slight negative skew (−1.13), but all the other variables met assumptions for parametric analyses. Women reported significantly higher paternal overprotection (*t*(224) = −3.24, *p* < .001) and higher affective empathy (*t*(224) = −5.87, *p* < .001) in comparison to men. In order to investigate the relative importance of each parent on male and female affective and cognitive empathy, we conducted a series of simultaneous, linear multiple regressions, separately for each sex. The four parenting variables (maternal and paternal care and overprotection) were entered as predictors, together with participant age as a control variable. Affective and cognitive empathy were entered as outcome variables. In order to control for the shared variance between the two components of empathy, the other component was entered as a predictor in each regression. The VIF values for all the variables were acceptable for regression analyses (all VIF < 1.80). Only the significant predictors are reported here, but readers are advised to contact the first author if they wish to obtain full results.

**Table 1:** Descriptive statistics and correlations for both sexes for parental care and empathy (men are reported above diagonal)

	Mean (SD) (men)	Mean (SD) women	1.	2.	3.	4.	5.	6.
1. Maternal care	27.38 (6.75)	26.75 (8.56)	−	−.57**	.30*	−.21	.14	.43**
2. Maternal overprotection	13.35 (7.60)	12.28 (7.55)	−.22**	-	−.17	.38**	.01	−.15
3. Paternal care	22.90 (8.50)	22.36 (10.00)	.44**	−.09	-	−.29*	.42**	.16
4. Paternal overprotection	8.59 (5.67)	12.06 (7.98)	−.18*	.38**	−.43**	−	.07	−.23
5. Affective Empathy	−.49 (.91)	.22 (.83)	.22**	.09	.12	.01	−	.35**
6. Cognitive Empathy	−.11 (.80)	.05 (.85)	.13	−.06	.02	−.05	.61**	−

*p < .05, **p < .01. Correlations are significantly different for men and women between maternal care and cognitive empathy (Fisher’s *z* = −2.81, *p* < .001) and paternal care and affective empathy (Fisher’s *z* = −224, *p* < 001)

For men, both paternal care and overprotection were significant predictors of affective empathy, and maternal care and paternal overprotection were significant predictors for cognitive empathy (Table 2). For women, maternal overprotection was significant predictor of affective empathy. Parental care variables were not significant predictors for any of the other affective or cognitive empathy variables in women.

**Table 2:** Regression models for cognitive and affective empathy for both sexes

Regression models	Predictor	*t*	*β*
Affective empathy in men	Maternal Care	−0.10	−.15
	Maternal Overprotection	−0.25	−.03
	Paternal Care	5.00	.48**
	Paternal Overprotection	2.23	.27*
*R* ^*2*^ = .35**			
Cognitive empathy in men	Maternal Care	3.60	.48**
	Maternal Overprotection	−1.52	−.11
	Paternal Care	−0.71	−.09
	Paternal Overprotection	−1.91	−.06
*R* ^*2*^ = .35**			
Affective empathy-women	Maternal Care	1.93	.15
	Maternal Overprotection	2.27	.16*
	Paternal Care	0.86	.07
	Paternal Overprotection	0.44	.03
*R* ^*2*^ = .42**			
Cognitive empathy -women	Maternal Care	0.32	.03
	Maternal Overprotection	−1.52	−.11
	Paternal Care	−1.10	−.09
	Paternal Overprotection	−0.81	−.06
*R* ^*2*^ = .40**			

*p < .05, **p < .01

## Discussion

We present the first study to show that recalled parental bonding in childhood predicts affective and cognitive empathy in adulthood, and this varies according to the sex of both parent and child. Affective empathy had a significant positive relationship with paternal care and overprotection in men, and significant positive relationship with maternal overprotection, and a non-significant positive trend with maternal care in women. Interestingly, high care and high overprotection form a so-called “affectionate control” style of parenting (Parker et al. 1979), which may have an important influence in affective empathy (see also Gao et al. 2010). The mechanisms behind the care, overprotection, and affective empathy remains to be investigated in future research.

Interestingly, affective empathy was related to recollections of care and overprotection by the same-sex parent. Humans may be predisposed to imitate own-sex behaviour (Losin et al. 2012), and have an increased identification with same-sex caregivers (Starrels 1994). Thus, the influence of the caregiving from the same-sex parent could be an important pre-requisite for the formation of affective empathy.

Developmental influences on cognitive empathy were substantially different to affective empathy, consistent with research identifying separate brain systems for each empathy type (Shamay-Tsoory et al. 2009). Maternal care related to cognitive empathy in men, but there was no relationship between recalled parenting and cognitive empathy in women. This finding supports previous research which implicated that maternal care is more important for the socio-emotional development of boys than girls (Etzion-Carasso and Oppenheim 2000), and has more substantial influence on perspective taking ability of boys (Laranjo et al. 2010). We extend this literature by providing the first evidence that the maternal care may have a long lasting effect on cognitive empathy in sons that persists into adulthood.

Further, the importance of maternal care for men’s (but not women’s) perspective taking may reflect overall sex differences in empathy and related abilities. In particular, women and girls typically display higher levels of cognitive empathy than men and boys (Baron-Cohen and Wheelwright 2004). Hence, male empathy has the greatest scope for development, and positive experiences such as high levels of maternal warmth and care may exert a greater influence. Indeed, in our study parental bonding exerted a greater overall impact on men compared to women. Additionally, the importance of maternal care may reflect the manner in which men and women interact with children. Whilst men are more likely to instigate physical activities that may promote motor skills such as physical coordination, women are more likely to promote social and pretend play (e.g., MacDonald and Parke 1986). These role play scenarios may encourage children to interact with others and adopt their perspective, which may have a long-lasting effect, influencing perspective-taking in adulthood as well.

Our results provide useful information for devising future intervention strategies aimed at improving empathy. Interventions have successfully increased empathy in children (Sahin 2012), adolescents (Castillo et al. 2013) and adults (Chaffin and Adams 2013). A more detailed understanding of those factors influencing the development of empathy may inform the design and delivery of these interventions. In the current study, all participants were raised with both parents present. Future research should consider empathy in adults who, as children, experienced little contact with same-sex parents, or were raised in one-parent families. In these environments, the opposite sex parent may exert a greater influence on empathy development. The potential influence of siblings and birth order in empathy in adulthood should also be investigated. Those raised with siblings may be frequently expected to consider the emotions of others (especially for elder siblings) and thus develop greater empathy. This is consistent with research indicating more advanced social skills and theory of mind in those with siblings compared to only children (Peterson 2000).

There are some limitations of the study. We used self-report measures, which may be susceptible to bias or inaccurate recall. A range of studies have, however, established the stability of the Parental Bonding Instrument over a 20 year period (Murphy et al. 2010; Wilhelm et al. 2005) and responses to the measure are not significantly influenced by current mood state (Lizardi and Klein 2005). Furthermore, perceptions of the parent-child relationship may be as important, or in some circumstances more important, than data collected directly from parents (Richman and Flaherty 1987). Nevertheless, it would be important to include more longitudinal designs investigating cognitive and affective empathy across the life-span, using a wider variety of methods in the research. In addition, there is a range of additional control variables which should be considered in a larger study including familial, social and other environmental factors. For example, prenatal testosterone exposure has been shown to be an important predictor of empathy in childhood (Chapman et al. 2006). It would be interesting to find how prenatal and childhood postnatal influences may interact with each other in affecting cognitive and affective empathy in adulthood.

To conclude, the current study indicates that adult empathy is influenced by parental bonding experienced as a child. The affective and cognitive components had different relationships to parenting variables, supporting the idea that these are partially different systems, and may be influenced by separate developmental trajectories. Affective empathy was influenced more by the same-sex parent, which could reflect the relationship between empathy and imitation, and our propensity to imitate same-sex individuals.
